# Feasibility of a low-cost hearing screening in rural Indiana

**DOI:** 10.1186/s12889-017-4724-7

**Published:** 2017-09-18

**Authors:** Khalid M. Khan, Sylvanna L. Bielko, Priscilla A. Barnes, Sydney S. Evans, Anna L.K. Main

**Affiliations:** 1 Department of Environmental and Occupational Health, School of Public Health, Indiana University, 1025 E Seventh Street, Room 025E, Bloomington, IN 47405 USA; 2 Department of Applied Health Science, School of Public Health, Indiana University, Bloomington, IN USA; 3 Department of Speech and Hearing Sciences, College of Arts and Sciences, Indiana University, Bloomington, IN USA

**Keywords:** Low-cost hearing screening, Noise-induced hearing loss, Occupational noise exposure, Rural health

## Abstract

**Background:**

Hearing loss remains a neglected public health issue in the rural and agricultural communities in the United States and therefore, promotion of a low-cost hearing screening may be important for these underserved populations. The major objectives of our study were to assess feasibility of a low-cost telephone-administered hearing test in rural Indiana and to identify the challenges, barriers and viable implementation strategies associated with this test. Also, we evaluated whether a focus group session could change the hearing health attitude of rural residents.

**Methods:**

We recruited 126 adults from six rural Indiana counties who participated in study activities in the following order: 1) a pre-focus group demographic, knowledge and attitude survey, 2) a focus group for discussing the feasibility of a telephone-administered hearing screening, 3) a post focus group attitude survey and 4) hearing was screened using an audiometer and self-assessment scale. These activities generated both qualitative and quantitative data, which were subsequently analyzed.

**Results:**

Hearing impairment was perceived as an important public health issue. Many participants expressed interests to try the low-cost National Hearing Test (NHT). However, participants recommended NHT to be facilitated by community organizations to provide access to landline phones. The focus group turned out to be an excellent awareness building activity producing significant improvement in hearing health attitudes. Comparison of self and audiometric evaluations indicated underestimation of hearing handicap in our rural study population.

**Conclusions:**

The study results underscore the urgent need for an effective strategy to promote low-cost hearing screening in rural US communities.

## Background

Adult hearing loss in the United States remains neglected as a public health issue [1]. In addition to the effects of age-related hearing loss in a progressively older U.S. population, noise exposure also results in noise-induced hearing loss [2]. Hearing loss may have detrimental effects on quality of life, communication, and routine activities [3], and may also be associated with impaired cognitive function, dementia [4], and all-cause mortality [5], as well as reduced social integration [1]. In the case of noise-induced hearing loss (NIHL), it may be possible to prevent further damage if caught early. Even negative side effects from age-related hearing impairment, such as depressive symptoms, loss of social interaction, and reduction functionality in the workplace [6, 7], could be mitigated by early intervention. Data from the National Health and Nutritional Examination Survey (NHANES) from 1999 to 2004 suggests immediate need for large-scale efforts to reduce modifiable risk factors and increase hearing screening to counter the problem [1]. Additionally, a report from an expert committee convened by The National Academies of Sciences, Engineering, and Medicine has prioritized the collection of population-based data on hearing impairment and hearing health care and improvement of access to hearing health care for underserved populations [8]. Although untreated hearing loss has negative effects on quality of life, only about 15–30% of those who could benefit from hearing assistive technology use it [9, 10].

While current literature has emphasized the prevalence of hearing loss in urban areas, it remains an equally significant problem in rural communities. Age-related hearing loss is common among all types of populations and NIHL has been associated with occupational noise exposure particularly among rural farmers [11]. Most occupations with hazardous noise exposure are regulated by the Occupational Safety and Health Administration (OSHA), but many farms are OSHA-exempt. In addition, many are small or family run and do not offer health awareness programs [11]. Due to this health disparity, rural farm workers may not have adequate knowledge on the risks associated with noise exposure or of the value of adequate hearing protection. The perception of hearing loss and hearing impairment may differ between rural farm workers and rural non-farm residents, due to the possibility that farm workers are regularly exposed to hazardous noise and may have been warned about its effects by other workers or workplace training.

Implementation of hearing screening programs in rural areas may be extremely challenging due to persisting health disparity issues. One of the main issues is lack of funding for health programs. Residents of rural communities earn less on average than those in urban communities and pay less in tax dollars, while the local public health officials have fewer resources to draw on for funding health related programs [12]. Individuals living in rural communities represent one-fifth of the total U.S. population, but only 10% of the number of U.S. physicians are available in rural health care centers [13]. At the same time, many rural communities have been struggling with other public health issues such as poverty, risky health behaviors, lack of health insurance, and limited access to health care [13]. A low-cost hearing screening test may be ideal as the first step for improving the hearing health of rural people.

Low-cost telephone administered screening tests utilizing three-digit sequencing presented in noise have been developed, validated and implemented in seven countries. Each of these tests have been based on a test protocol conceived by Smits and colleagues in the Netherlands [14]. More recently, the U.S. version of a landline phone technology-based screening test, known as the National Hearing Test (NHT), has been developed by hearing scientists from Communication Disorders Technology, Inc. (CDT), Indiana University, and their collaborators at VU University, Amsterdam [15]. The telephone test incorporates background noise and uses signal-to-noise ratio (SNR) criteria to categorize each person being tested as “within normal range,” “slightly below the normal range,” or “substantially below the normal range” [16]. When SNR thresholds were compared with corresponding pure-tone threshold in a recent study, evidence was obtained that showed the NHT to be sufficiently sensitive and specific for use as an auditory function screening protocol [17]. This validation study was conducted on military veterans and non-veterans. What is not known is whether NHT can reproduce the same results, in terms of validity, in rural areas where both age-related and noise-induced hearing loss are prevalent.

In order to address the use of a low-cost hearing screening test and hearing health in rural communities, we addressed several specific aims in this study. The first aim of the study was to assess the acceptability of a low-cost hearing screening test. In order to evaluate the feasibility of the test, we asked the participants questions about the following: 1) how important hearing is in rural communities, 2) if they are willing to take the test, 3) identifying the best mechanism in which the test can be implemented across rural communities, 4) barriers and obstacles to the test and taking the test, and 5) evaluation of the test by the test takers. The second aim of the study was to determine whether the discussion and education on hearing health could have a short term impact on attitude towards hearing health. The third aim of the study was to evaluate whether hearing health knowledge levels are different between the general population and those exposed to loud noise through their occupation. The last aim was to examine the correlation of three different hearing screening tests (i.e. self-report, pure-tone audiometric screening, and NHT).

## Methods

### Study area and population

The study was conducted in six counties of southern Indiana with the help of Indiana Rural Health Association (IRHA) who have long-standing collaborations with many community organizations such as community service centers, churches, hospitals and clinics located in rural Indiana. An initial list of 10 counties of southern Indiana was generated by the staff of IRHA who assisted the researchers of Indiana University-Bloomington School of Public Health (IUB-SPH) to recruit study participants. Counties in this list met the following criteria to be considered as a unit of the study area: 1) had one or more rural community organization (RCO) that previously partnered with IRHA in a public health project, 2) the partner community organization had contact information of at least 50 local residents including individuals who had a history of working in noisy environment (e.g. farmers) in their lifetime, and 3) the facility that the partner organization was able to provide for the study would include separate rooms to concurrently conduct focus group and hearing screening sessions. Six counties Daviess, Greene, Lawrence, Orange, Owen and Sullivan were randomly selected from the initial list for recruiting participants. The study was approved by Indiana University Human Subjects Office, which is the administrative office that supports the Indiana University Institutional Review Boards (IRB) in April 2015.

### Recruitment and data collection

In the spring of 2015, IRHA staff communicated with RCO and finalized a date for each county to conduct all necessary study activities. Subsequently, an RCO representative contacted the potential participants, checked his/her availability and eligibility, and then briefly described the study procedure. Recruitment announcement was posted in the RCO’s notice board at least 7 days prior to the study session. Additionally, the RCO representative made a direct phone call to a potential participant whenever a contact phone number was available. IRHA and RCO staff jointly maintained a list of potential participants who initially responded positively to participate in the study session and gave them a reminder about the schedule of the study activities 48 h before the study session. On the day of the study session, written informed consent was obtained from 126 adult participant from the six counties by the researchers from IUB-SPH. Study activities started with a short demographic survey questionnaire that was filled out by the participant. This was used to collect information about age, sex, ethnicity, occupation, income, housing status, history of occupational noise exposure, history of hearing screening and evaluation of hearing health status by an audiologists and use of hearing aids. This was followed by several other data and information collection activities as per the specific aims of the study: 1) short hearing knowledge questionnaire, 2) a pre-focus group attitude questionnaire, 3) focus group on the feasibility of a telephone hearing screening test, 4) self-evaluation of hearing of the participant using a questionnaire, 5) a post-focus group attitude test, and 6) an audiometric screening of hearing. All participants also received necessary information (including an access phone number and an access code) to participate in the telephone hearing screening known as the National Hearing Test (NHT) using a landline phone within 2 weeks of the study session.

### Outcome assessment

#### Hearing health knowledge

Participants filled out a 15-item hearing health knowledge questionnaire with each question presenting two possible responses: “TRUE” and “FALSE”. Questions were adapted from a hearing health public awareness publication from the World Health organization (WHO) [18] and a hearing conservation study [19]. Questions covered topics such as importance of hearing screening, importance of cleaning and treating ears, overexposure to chemicals and noise, and consequences of diagnostic delay.

#### Hearing health attitude

A 15-item attitude and belief questionnaire was adapted from a health belief model-based questionnaire, which was found to be a valid tool for assessing attitude in a previous US study [19]. Each item, a Likert-type statement, assessed hearing health attitude and belief of the respondent in one of the six categories, such as perceived susceptibility, severity, benefits, barriers, self-efficacy, and cues to action [20]. Response options on a 5-point scale (0–4) ranged from strongly agree (4 points) to strongly disagree (0 points) with neutral (neither agree nor disagree) in the middle. All participants completed this attitude questionnaire at the beginning of the study session (pre-focus group) and once again towards the end of the session (post-focus group) after completing all major activities including the focus group, hearing health self-assessment, and audiometric hearing screening.

#### Self-evaluation of hearing

All participants completed a 10-item screening version of the Hearing Handicap Inventory for Adults (HHIA-S) [21]. Five items of the HHIA-S are categorized as emotional and remaining five are categorized as social/situational. All items are selected from the 25-item version of the HHIA. Each item had three possible responses. Participants received 4 points when the response was “yes”, 2 points when the response was “maybe” and 0 points when it was “no”.

#### Audiometric hearing screening

Pure-tone air conduction screening was conducted by a study research assistant who was trained by an audiologist. All screenings were conducted in a quiet, private room with limited external influences (i.e. a personal office within a church). Screening was performed on both left and right ears using the Beltone 10D portable audiometer at the frequencies of 500, 1000, 2000, and 4000 Hz at a level of 25 dB HL. The participant was instructed to respond positively by raising their hand each time they heard the tone. If a participant had one “no response” at any frequency in either ear they “failed” the screening and were subsequently advised to visit an audiologist for additional hearing evaluation.

#### Focus groups

The purpose of the focus groups was to explore hearing health related attitudes and beliefs of adults living in rural Indiana, including both those who have been exposed to loud noises from occupational activities and those who have not. In addition, perceptions about the NHT were examined in order to assess use and identify possible organizations or groups to administer it.

An interview guide was developed to elicit attitudes and beliefs about the importance of hearing in comparison to other aspects of health; assess good, bad, easy, and difficult aspects of getting a hearing test; and explore willingness to participate in a telephone hearing test. A member of the research team moderated each focus group.

A total of six focus groups were conducted with farmers and non-farmers residing in rural communities. Five focus group sessions were audiotaped and transcribed; one group was not taped due to equipment failure. Participants’ names were not included in the transcripts to maintain confidentiality. For this reason, frequencies for statements and opinions made in the groups were not calculated.

### Statistical analysis

Qualitative data analysis was conducted using Dedoose software version 7.1. A member of the team reviewed transcripts. Descriptive and in vivo coding were used to summarize a word or a phrase capturing participants’ feelings and attitudes about hearing and taking hearing tests as well as factors that prompt or hinder promotion of hearing health in rural communities. Codes were grouped into categories. Analytic/memo writing was as a technique used to make connections between categories and to further identify patterns and themes.

Quantitative data analysis was conducted using SPSS® 24. Study participants were divided into two groups: the first group with no history of significant occupational noise exposure and the other group with history of occupational noise exposure. The two groups were then compared for the differences in sociodemographic characteristics using chi-squared test for categorical variables and independent sample *t*-test for continuous variables. To indicate internal consistency and reliability of the attitude items in the questionnaire, we calculated Cronbach’s alpha for pre and post attitude scales separately***.*** Participants received 1 point for each correct answer in the knowledge questionnaire. Points were then added up to compute a summary knowledge score. Pre- and post-attitude summary scores were also calculated by adding points (on a Likert scale 0–4) for each of the 15 items. A paired *t*-test was used to examine the difference between pre- and post-focus group attitude scores to examine if the attitude changed significantly within each group of participants.

## Results

### Participant characteristics

All participants, aged 25 and over, were recruited from rural communities located in six counties (Daviess, Sullivan, Greene, Owen, Orange, and Lawrence) of the state of Indiana. Participants who reported high-noise exposure in their present or any previous job (i.e. those who worked at jobs that involved noisy tasks or jobs for which hearing protection was recommended) were classified as “potential occupational noise exposure,” whereas those who did not work in such environment were classified as “no occupational noise exposure”. The potential noise exposure group was significantly older, had significantly higher proportion of males and lower level of education than the group consisting of participants with no history of occupational noise exposure (Table 1). The majority (56.9%) of participants in the potential occupational noise exposure group had >30 years working in high noise environments.

**Table 1:** Demographic data comparing participants with potential occupational noise exposure with those without occupational noise exposure

Variables	Potential occupational noise exposure (*n* = 59) Mean (SD) or n (%)	No occupational noise exposure (*n* = 67) Mean (SD) or n (%)	t or Chi-Square values	*P* values
Average age (yrs)	62.0 (13.6)	55.8 (14.2)	2.5	0.01*
Gender (% males)	36 (61.0)	18 (26.9)	14.94	< .001*
Ethnicity (White/Non-Hispanic)	57 (96.6)	66 (98.5)	0.49	0.49
Marital status (Married)	48 (81.4)	49 (73.1)	2.93	0.49
Education level				
Less than high school	4 (6.8)	3 (4.5)		.01*
High school	22 (37.3)	27 (40.3)		
Technical	9 (15.3)	1 (1.5)	14.93	
Undergraduate degree	21 (35.6)	22 (32.8)		
Graduate degree	3 (5.1)	14 (20.9)		
Years working in high noise				
0–10 years	12 (20.3)	NA	NA	NA
11–20 years	8 (13.6)			
21–30 years	10 (16.9)			
> 30 years	29 (49.2)			

*indicates significant *P* value

### Focus group findings

The major finding from the focus group sessions are presented in Table 2. Participants expressed their views and opinions in three different areas of hearing health such as the importance, feasibility of a low-cost hearing test, and potential facilities where the test could be conducted. When participants were asked about recent hearing screening, several individuals reported received hearing screening through their job, especially if they were employed by a governmental entity, while others had not been tested in as long as 10–15 years. Older farmers reported not using protective gear because it was difficult to do their job efficiently. Many people felt not much could be done to address hearing related issues among older farmers, and that more attention should be given to younger farmers or young people who are around loud noises.

**Table 2:** Focus group findings on hearing health issues

Major Research Question	Identified as benefits (positive factors)	Identified as barriers (negative factors)
1. Hearing as an important factor to health	1. Hearing perceived as being important to very important. 2. Hearing health practices have evolved (testing and hearing protection have improved over time)	1. Many participants reported not having a recent hearing test. Some had not had a test in as long as long as 10–15 years ago 2. Although participants viewed hearing as important, they felt that hearing was not a major concern when compared to other health issues (i.e. heart disease, cancer, and diabetes) 3. Difficult to find time to get hearing tested 4. Hearing was perceived as being an issue that people could “adjust” to (i.e. you could increase the volume on the television and cell phones or have family members speak louder so they can hear them) 5. Older farmers reported not use hearing protective gear because it makes it difficult to do their job efficiently
2. Exploring feasibility of taking the National Hearing Test (NHT)	1. Most individuals shared positive but mixed feelings about taking the NHT (i.e. would take it out of curiosity but were interested in how the test differed from the pure-tone audiometer screening)	1. Participants do not have a landline phone at home 2. Several participants wore hearing aid or were already aware of their hearing loss and felt the NHT would not be helpful 3. Some participants thought a more rigorous test is needed (i.e. pure-tone audiometer screening) 4. From farmers who have not been diagnosed with a hearing issue, many felt that providing protective gear would be more beneficial than screening test
3. Community space to disseminate NHT and Hearing Health Strategies	1. Participants would consider taking NHT if offered and highly promoted in the community 2. Appropriate venues for offering NHT: - Middle and high schools - Hospitals - Community-wide health fairs - Libraries - Fraternities and sororities with hearing protection as primary focus	1. Participants were not willing to pay for testing

Participants mentioned that individuals may not want to accept that there may be a problem. It is easy to ignore. One participant stated:

*I think there are more people wearing [hearing aids] now than they use it because they are handier…and as far as the expense…I don’t think it is the expense of the hearing test. It is the experience of the hearing aid. If you do find out that you loss…[your hearing].*

Some individuals in the focus group noted that they noticed certain signs such as ringing in the ear. Most of the time, a spouse, friend, or co-worker suggested that they go get a hearing test. Few followed up on the recommendations while others failed to do so.

Most individuals shared positive, but mixed feelings about taking the NHT. A few individuals felt that the NHT should be directly towards a younger population, specifically middle and high school students. Additionally, a number of older participants felt loud music played through earphones or through the speakers of a vehicle were more of a concern and could be quickly remedied using the NHT.

The focus groups not only assisted researchers’ answer specific study aims, they also served as a health awareness intervention for the majority of participants. Personal stories were shared among participants about hearing tests, hearing aids, recreational noise exposure, and community norms and expectations of hearing. One participant mentioned:

*I think [name of focus group participant voicing important of education] is right about pushing it through education. Prior to coming here, I still would have ….continued to not use hearing protection for certain things like doing hunting….*

Although this participant works in an environment where exposure to noise is low, the discussion encouraged her to think about spaces where she is exposed to higher levels of noise. Recreational activities, such as motor biking, sports shooting, concerts, and hunting were discussed as spaces where hearing protection is needed. The focus group discussion prompted her to revisit their own beliefs. In addition, the focus groups prompted potential health strategies to raise awareness about hearing health. Public health awareness was most frequently cited. One participant stated:

*I think they need to do public service announcements like they do for smokin’. On television. Cuz people…we can tell them and it won’t do any good, but we need somebody to tell them about noise…what to listen to…why they can’t hear….especially like for they do for people who are smokin’.*

Other strategies included enforcement of workplace policies to encourage use of hearing protection and health education and science curricula in schools.

#### Attitude

**Fig. 1:**
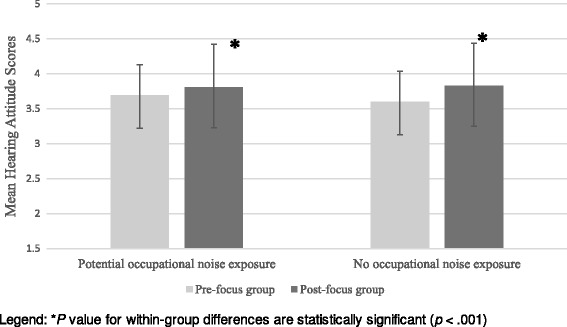
Comparison of pre- and post-focus group hearing attitude scores for both groups

The mean hearing attitude scores significantly increased after the participation in the focus group among participants with occupational noise exposure (*t* = 1.34, *P* = 0.03) and without occupational noise exposure (*t* = 4.14, *P* = 0.001) (Fig. 1). However, there was no significant differences for the change of pre- to post-focus group attitude scores between the two exposure groups (*t* = 1.34, *P* = 0.19). The attitude scale used in the study was found to be extremely reliable as we observed high Cronbach’s alpha values for both pre (Chronbach’s α = 0.79) and post (Chronbach’s α = 0.89) attitude and belief questionnaire. The mean attitude score for the entire sample (*n* = 126) increased from 3.64 (SD 0.46) to 3.83 (SD 0.59).

#### Knowledge

There was a significant difference for knowledge scores (mean % correct) between the participants with potential occupational and no occupational noise exposure (*t* = 2.34, *P* = .020). The no occupational noise exposure group has a significantly higher knowledge score (71.3% ± 21.4%) than the potential occupational noise exposure group (62.2% ± 21.1%).

#### Screening and assessment for hearing

Results from three different hearing screening and assessment conducted on the participants found rural Indiana adults underreporting their hearing impairment through HHIA-S questionnaire in both groups. However, the misclassification of hearing ability was significantly higher in the group with no occupational noise exposure (40.3%) than the group with potential occupational noise exposure in their lifetime (22%) (Chi-Square = 4.83, *p* = 0.03) (Table 3). Audiometric screening identified almost 90% of the participants in the potential occupational noise exposure group having hearing impairment in at least one of the two ears, which was lower in the other group (i.e. about 66%). When we observed the self-reporting of hearing handicap by the participants the percentage of hearing handicap dropped down to about 71% and 52% in potential occupational and no occupational exposure groups, respectively. The majority of both groups’ self-reported hearing handicap in both groups fell within mild to moderate score range (HHIA-S = 10–24).

**Table 3:** Comparison of results from two screening tests (i.e. audiometric screening and HHIA-S) between the groups

	*Potential occupational noise exposure* *(n = 59)*		*No occupational noise exposure (n = 67)*	
	Self-reported Hearing Handicap (HHIA-S ≥ 10)% (n)	No Hearing Handicap reported (HHIA-S < 10)% (n)	Self-reported Hearing Handicap (HHIA-S ≥ 10)% (n)	No Hearing Handicap reported (HHIA-S < 10)% (n)
Failed audiometric screening test	69.5% (41)	20.3% (12)	38.8% (26)	26.9% (18)
Passed audiometric screening test	1.7% (1)	8.5% (5)	13.4% (9)	20.9% (14)

Out of 126 participants, only 23.8% (*n* = 30) completed the low-cost NHT within 2 weeks of the focus group and two other hearing screening events. Participants who had potential occupational noise exposure had higher NHT participation rate compared to those who had no occupational noise exposure history (27.1% vs 20.9%).

## Discussion

Participants of our study who lived in rural counties of Indiana recognized hearing as a major public health problem, even though lack of a landline telephone in homes diminished their interest in trying out the affordable telephone-administered hearing test. A major finding of the study was that rural residents identified several community resources and organizations as the trustworthy providers of this specific hearing screening, even if they charge a low price for this service. The NHT has been introduced to US urban populations in recent years achieving remarkable success. During a two-month promotional period in mid-2014 when the test was offered free-of-cost, about 31,000 individuals took the NHT with majority having perceived hearing problem in at least one ear [22]. Several unique characteristics such as high scientific validity, confidentiality of the screening results (informed only to the test taker) and no commercial relation to service providers or manufacturers of hearing aid made the test very successful in urban populations [22]. Data from our study also indicate that the success of NHT in rural US communities may rely on the presence of a community-based organization where the test can be administered on a landline-phone even for a low cost. Additional information dissemination regarding the benefits of NHT via newspaper reports and blogs may also help to some extent as evident in the recent urban study [22].

When we compared the hearing screening audiometric and self-assessment outcomes by two groups of participants, we observed a higher misclassification rate among the participants who had no history of occupational noise exposure. Overestimation of hearing loss was very minimal in the occupational noise exposure group but was noticeable (13.4% group participants) in the group with no occupational noise exposure. This may be attributable to a difference in perceived hearing between groups as individuals with a history of occupational noise exposure tend to consider hearing loss as a natural and unavoidable health consequence in later stages of life [11]. We do not know exactly what other factors might have contributed to the difference in misclassification and underestimation, however, literature indicates that a combination of demographic factors such as age, gender and education are associated with over and underestimation of hearing loss when self-assessment of hearing is used [23].

There are several limitations of the study. The study did not capture information about past recreational noise exposure such as recreational firearm use, which was shown to be associated with hearing loss [24]. Information on this issue might have been useful in explaining the differences in over and underestimation of hearing loss between the two groups. We were also unable to compute correlations between audiometric, self-screening and NHT outcome variables. Our audiometric screening method produced a binary outcome (i.e. pass or fail for the screening) instead of pure-tone thresholds. However, it is already known from a previous study that the NHT outcomes and mean pure-tone thresholds all correlated significantly (*P* < 0.001) with the highest correlation observed at 2000 Hz [16].

Our study was conducted in Indiana, where around 14% and 24% of the population resided in the rural counties and semi-rural counties according the 2010 census [25]. In general, residents of rural counties across the United States have limited access to hearing healthcare facilities, and hearing loss is a neglected public health problem in these economically disadvantaged populations [26]. Compared to urban populations their rural counterparts have lower education and household income and greater travel distance to hearing healthcare center creating delays in treatment and resulting in significant hearing health disparities [26]. Therefore, hearing healthcare in rural communities require more low-cost easily implementable screening, which could potentially motivate the hearing handicapped rural residents to seek a full evaluation by an audiologist.

While the NHT is a valuable resource to offer low-cost hearing screening in rural communities, there are several potential barriers that need to be addressed. The NHT was developed to be administered via landline telephone [15, 22] and should not be taken on a cellular phone. Participants shared that many did not have a landline telephone, which would make it difficult to complete the test from home. Participants also said that they would consider taking the NHT if offered and highly promoted in the community but were not willing to pay for it. Even with the NHT being offered at a low-cost (approximately $5), the participants believed that there are other chronic health issues (such as diabetes and cardiovascular disease) that should be prioritized over hearing health, which they could adapt to by having people speak louder. These barriers could be mitigated by having community facilitators (i.e. schools, churches, hospitals/clinics, community-wide health fairs, etc.) provide the test on site via a landline. This would require a quiet room with a landline and the provision of an access code for the participants. Community involvement and available facilitators are necessary for the NHT to be feasible in rural communities.

## Conclusions

Our study indicated that adults in rural Indiana perceived hearing loss as an important public health challenge for rural populations and were enthusiastic to take the low-cost telephone-based National Hearing Test (NHT). However, due to the lack of landline phone access in a majority of the households our study participants suggested that community organizations could play the role of trusted facilitators by providing the logistics required for this screening. Results from the focus groups and other formats of hearing screening (e.g. questionnaire and audiometric screening) reflect the fact that in spite of the ignorance and underestimation of the hearing health issues in our study areas, education and awareness building activities coupled with the low-cost NHT could bring marked improvement in hearing health status of rural US populations.

## Abbreviations

CDT: Communication Disorders Technology, Inc.
HHIA-S: Hearing Handicap Inventory for Adults
IRB: Institutional Review Boards
IRHA: Indiana Rural Health Association
IUB-SPH: Indiana University-Bloomington School of Public Health
NHANES: National Health and Nutritional Examination Survey
NHT: National Hearing Test
NIHL: Noise-induced hearing loss
OSHA: Occupational Safety and Health Administration
RCO: Rural community organization
SNR: Signal-to-noise ratio
WHO: World Health organization
